# Review: Use of Electrophysiological Techniques to Study Visual Functions of Aquatic Organisms

**DOI:** 10.3389/fphys.2022.798382

**Published:** 2022-01-27

**Authors:** Xiaolong Gao, Shihui Lin, Mo Zhang, Mingxin Lyu, Yafeng Liu, Xuan Luo, Weiwei You, Caihuan Ke

**Affiliations:** ^1^State Key Laboratory of Marine Environmental Science, College of Ocean and Earth Sciences, Xiamen University, Xiamen, China; ^2^Fujian Key Laboratory of Genetics and Breeding of Marine Organisms, Xiamen University, Xiamen, China; ^3^College of Life Science and Technology, Huazhong University of Science and Technology, Wuhan, China

**Keywords:** aquatic animals, electroretinogram (ERG), visual sensitivity, photoreceptor, diel rhythm

## Abstract

The light environments of natural water sources have specific characteristics. For the majority of aquatic organisms, vision is crucial for predation, hiding from predators, communicating information, and reproduction. Electroretinography (ERG) is a diagnostic method used for assessing visual function. An electroretinogram records the comprehensive potential response of retinal cells under light stimuli and divides it into several components. Unique wave components are derived from different retinal cells, thus retinal function can be determined by analyzing these components. This review provides an overview of the milestones of ERG technology, describing how ERG is used to study visual sensitivity (e.g., spectral sensitivity, luminous sensitivity, and temporal resolution) of fish, crustaceans, mollusks, and other aquatic organisms (seals, sea lions, sea turtles, horseshoe crabs, and jellyfish). In addition, it describes the correlations between visual sensitivity and habitat, the variation of visual sensitivity as a function of individual growth, and the diel cycle changes of visual sensitivity. Efforts to identify the visual sensitivity of different aquatic organisms are vital to understanding the environmental plasticity of biological evolution and for directing aquaculture, marine fishery, and ecosystem management.

## Principles of Visual Electrophysiology

The retina is an important tissue that perceives external optical information and converts it to visual information. In vertebrates, after entering the eye, light passes through the refractive medium in front of the retina before reaching the retina. The photoreceptor cells at the outer end of the retina convert light photon signals into electrical signals, which ultimately form visual signals through integrative processing using bipolar cells, optic ganglion cells, horizontal cells, and amacrine cells. These visual signals are transmitted to the optical center via the optic nerve to produce vision (Evans and Browman, 2004; Lorber et al., 2016).

**FIGURE 1:**
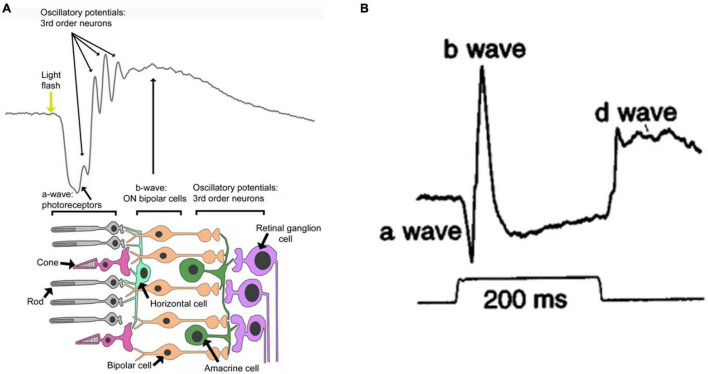
**(A)** Schematic representation of a flash electroretinogram (ERG) and the retinal events contributing to it (Cameron et al., 2008). **(B)** Primate photopic ERG responses to 200 ms stimuli (Bush and Sieving, 1996).

Using a specific electronic instrument, electroretinography (ERG) records electrical potential changes in the retina when it is subjected to light stimuli. Flash ERG is commonly described in terms of several discrete components (Figure 1B). Under light stimuli, the retina initially produces a small negative wave (also referred to as the a-wave). If the intensity of light stimuli is large enough, an early receptive potential (ERP), composed of two oppositely polarized waves, will appear before the a-wave. This is followed by a positive b-wave and a set of rhythmic wavelets with higher frequencies and lower amplitudes superimposed on the rising branch of the b-wave (also referred to as oscillating potentials). A positive wave with slower rise, called a c-wave, also occurs. As the light stimulus ends, a positive upward protrusion, or d-wave, can be detected. All these waves are derived from different components (i.e., cells) of the retina (Figure 1A). The a-wave is mainly derived from photoreceptor cells; light absorption by these cells triggers conformational changes of photosensitizing pigments, thereby triggering the cascade of G proteins and closure of the cGMP-activated cation channel, which reduces the influx of Na^+^ and Ca^2+^ and leads to hyperpolarization of the membrane (Graham and Klistorner, 1998). The b-wave is primarily associated with the activity of depolarized bipolar cells (Pardue and Peachey, 2014). The c-wave consists of the superposition of two oppositely polarized potentials; one is the positive potential resulting from hyperpolarization of the retinal pigment epithelium apical membrane, and the other is the negative potential resulting from Müller glial cells (Tarchick et al., 2016). The d-wave is an off-response associated with the activity of photoreceptors and Müller cells (Ueno et al., 2006). The ERP originates from the outer segment of the photoreceptor and results from intramolecular electron transfer, which is triggered by changes in the configuration of visual pigment molecules during light stimuli (Fei and Fang, 1989). Oscillatory potentials are related to the action between bipolar cells, amacrine cells, and ganglion cells in the inner retina. The amplitude of a–d waves varies with the duration and intensity of light stimuli and the adaptive conditions of the retina. However, the b-wave is a positive phase response that is most sensitive to variations of external factors, thus it can indirectly reflect the activity of photoreceptors. Therefore, the amplitude of the b-wave is often treated as the response amplitude of a set of light stimuli (Brown, 1969; Rager, 1979; Gacic et al., 2014; Hayasaka et al., 2021).

## Milestones

The use of ERG dates back more than 100 years. Holmgren (1865) was the first researcher to use a galvanometer to connect electrodes to the front and rear of the eyeball. He was able to measure a rapid current change when an eyeball isolated from a frog was subjected to light stimuli, with the current appearing positive on the corneal side. Through subsequent experiments, Holmgren (1870), found that after an eyeball isolated from an animal was transversely cut, the potential difference was also measurable from the vitreous side to the posterior sclera, and a change in potential also occurred during light stimuli. This result indicated that the retina was the source of the electrostatic potential of the eye. Kühne and Steiner (1880) detected a change in potential in the retina isolated from the eyeballs of animals, but not in the eyeball without the retina, supporting the premise that the retina was the source of the signal.

The components and origins of ERG signals have been studied extensively. As observed in previous studies, Gotch and Francis (1903) reported that a more rapid negative potential occurred before the positive potential. Ishihara (1906) described a slowly rising positive wave following the appearance of the positive potential on the ERG. Einthoven and Jolly (1908) referred to the first negative wave as an a-wave, the positive wave that occurred subsequently as a b-wave, and the wave that occurred after the cessation of light stimuli as a d-wave. They further indicated that these waves might originate from the superposition of two components. However, referring to all components of the ERG profile, Granit (1933) proposed the “three waveguide range.” He argued that the ERG signal originated from superimposition of three components (PI, PII, and PIII) and inferred that PI was a type of c-wave, PII was a type of b-wave, and PIII was associated with both a- and b-waves. Adrian (1946) reported double b-waves and referred to the first as a photopic b-wave and the latter as a scotopic b-wave. He showed that the photopic b-wave was associated with cone cells and the scotopic b-wave was associated with rod cells. Riggs and Johnson (1949) found that some small amplitude waves were superimposed on the b-wave as measured by ERG. Heck and Rendahl (1957) identified four peaks of these small amplitude waves, and Yonemura et al. (1962) referred to these small amplitude waves as oscillatory potentials. Dawson et al. (1968) reported that the shape and quantity of oscillating potentials depended on the experimental conditions. Brown and Murakami (1964) described a wave with almost no latent period under strong light, which he referred to as the ERP.

The progress of microelectrode intracellular recording techniques promoted further studies of the origin of ERG components. Murakami and Kaneko (1967) recorded two PIII components using a step-by-step recording technique and named them distal PIII and proximal PIII, respectively. They also proposed that a-waves originated from the synapses of the photoreceptor cells. Based on microelectrode recordings in cells, Miller and Dowling (1970) found that the light-induced action potential of Müller cells mostly coincided with b-waves. Subsequent studies revealed that the potential changes of Müller cells were triggered by an increase in the concentration of extracellular potassium ions, due to the depolarization of retinal neurons, instead of being directly induced by light, and that the generation of b-waves reflected the light-induced depolarization of ON bipolar cells (Stockton and Slaughter, 1989; Gurevich and Slaughter, 1993; Hood and Birch, 1996). In addition, the activation of Nav channels on cone bipolar cells and rod bipolar cells affects b-waves measured using ERG (Mojumder et al., 2008; Smith et al., 2013).

The progress of ERG research is closely related to the development of instruments. The ERG profiles of aquatic animals are usually recorded with a metal wire placed on the corneal surface or a glass electrode penetrating the eye. Cotton core electrodes (Northmore and Muntz, 1970; Matsuda and Wilder, 2010) and metal electrodes (Horodysky et al., 2013; Kopperud and Grace, 2017; Warrington et al., 2017; Kingston et al., 2019) can also be used. After the 1960s, glass microelectrodes began to be used in numerous applications (Karita et al., 1973; Clark, 1975; Mccormick et al., 2019; Venkatraman et al., 2020; Hayasaka et al., 2021). Metal electrodes are generally made of platinum or tungsten rods, silver wires, or silver-silver chloride (Hanyu and Ali, 1964; Yang et al., 1990), whereas glass microelectrodes are generally filled with ionic solutions (e.g., KCl solution) and then connected to an external metal wire. Other kinds of electrodes, such as stainless steel electrodes (Fuentespardo et al., 1997) and polyethylene pipe steel electrodes (Weber, 1982), are less commonly used. To date, tungsten halogen lamps (Weber, 1982), xenon lamps (Karita et al., 1973; Clark, 1975; King-Smith and Cronin, 1996), and light-emitting diodes (LEDs) (Vetter et al., 2019; Hasenei et al., 2020) are the main light sources used in ERG studies, and the light intensity and wavelength range are controlled by neutral filters and interference filters.

## Application of Electroretinographys in Studies of Vision in Aquatic Organisms

**FIGURE 2:**
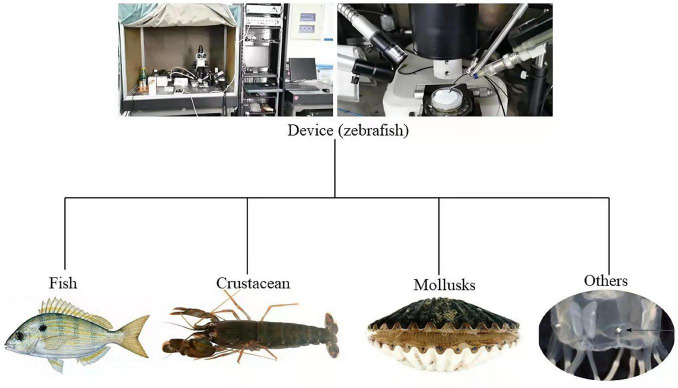
Part of the device diagram for electroretinogram testing of aquatic animals discussed in this review (Coates et al., 2006; Mccomb et al., 2013; Kingston et al., 2017, 2019; Liu et al., 2021).

For the majority of aquatic organisms, vision is crucial for predation, hiding from predators, communicating information, and reproduction (Johnsen, 2005; Mccormick and Allan, 2016; Butler et al., 2019). Even slight changes of light intensity and spectral composition may impact the feeding, survival, and growth of aquatic organisms (Villamizar et al., 2011). Efforts to measure the visual sensitivity of aquatic organisms help us to assess the effects of light conditions on the growth and development of cultured species. The reliance of aquatic animals on color vision and visual sensitivity must be considered when constructing fish facilities and improving fish yield efficiency (Browman, 2005). Therefore, electrophysiological data can be used to describe the visual sensitivity of aquatic organisms (Figure 2), which in turn provides direct data relevant to aquaculture, marine fishery, and ecosystem management (Hasenei et al., 2020).

Visual sensitivity includes spectral sensitivity, luminous sensitivity, and temporal resolution as well as contrast and polarization sensitivities. The b-wave amplitude of an electroretinogram indicates the response of the retina to a variety of light stimuli, and luminous sensitivities can be expressed by two indicators: K_50_ [the irradiance required to generate 50% of the peak amplitude (V_ma_x)] and dynamic range (the irradiance difference required to generate 5% and 95% of the V_max_). Spectral sensitivity can be expressed as the reciprocal of the irradiance required to generate the standard reaction. Temporal resolution is a measure of the integration time of the eye, which can reflect the ability of an organism to track moving objects. The highest frequency at which the signal produced by the eye and the light stimuli of the set irradiance retain the same phase is defined as the critical flicker-fusion frequency (CFF) (Hayasaka et al., 2021). Studies of visual sensitivity often involve separating the photoreceptor cell cone from the rod signals, and rods and cones can be distinguished according to their characteristics. The rod is sensitive to dark light and light at higher frequencies, whereas the cone is sensitive to bright light and light at lower frequencies. During adaptation to darkness, the sensitivity of the rod to light is 1,000 times greater than that of the cone. Rods can be bleached by bright light, so that use of dark-adapted blue light stimuli can separate the rod from the cone signals. In addition, adaption to different light spectral components diminishes the function of cones, which allows differentiation of different types of cones. As an alternative method, the two types of photoreceptor cell signals can be separated by temporal resolution, and high frequency flicker stimuli can accurately separate cone cell signals (Fei and Fang, 1989; Cameron et al., 2008).

### Application of Electroretinography in Studies of Fish Visual Functions

#### Types of Photoreceptors

Photoreceptors are the retinal cells that convert light (photon) signals into electrical signals to propagate photoelectrical conversion. Vertebrate photoreceptors are classified into cone and rod cells. The outer segments of rod cells are rod-shaped and contain rhodopsin, which is sensitive to light and mainly responsible for dark vision. The outer segments of cone cells are conical and are responsible for photopic vision and color vision, and different cones have opsins that are sensitive to different wavelengths.

The zebrafish, *Danio rerio*, has four types of cone cells: ultraviolet wavelength, short wavelength, medium wavelength, and long wavelength. When the CFF of *D. rerio* was measured by ERG, no signal was detectable at 3 days post-fertilization (dpf); signals appeared at 4–12 dpf, but the amplitude was lower than that of adults and there was no response under low light intensity. The CFF-light intensity function curve at 15–24 dpf was similar to that of adult zebrafish, which was consistent with morphological data. Thus, these results suggested that photoreceptors began to develop at 2 dpf and that complete rod cells first appeared at 12 dpf (Branchek, 1984; Branchek and Bremiller, 1984). Variations of spectral sensitivity were observed in *D. rerio* under dark adaptation during development, and the spectral sensitivities at 6–8 and 13–15 dpf were due to cone cells, with very little contribution by rod cells. Measurement of the spectral sensitivities of the retina at 21–24 and 27–29 dpf showed that both rod and cone cells were functioning. The spectral sensitivity of *D. rerio* adults was mostly due to rod cells and ultraviolet-type cone cells (Bilotta et al., 2001).

Using the b-wave amplitude of the electroretinogram as an indicator of the response to light stimuli, Lu et al. (2021) analyzed the spectral sensitivity of *D. rerio* adults under dark adaptation. They identified spectral sensitivity peaks at 500 and 365 nm. After cone and rod cells were separated under photopic and scotopic conditions, the b-wave amplitude of *D. rerio* increased with age under dark adaptation with the same light stimuli. However, under light adaptation, no significant change in the b-wave amplitude was detected in any age group (Nadolski et al., 2020). Brockerhoff et al. (2003) discovered a cone mutant of *D. rerio* called *no optokinetic response f*, which had impaired cone function and normal rods. Electroretinography results confirmed the lack of an ERG response when the mutant was under light adaptation. Under dark adaptation, the b-wave amplitude was consistent with that of wild-type fish. Compared with wild-type *D. rerio* juveniles at 6 dpf, the mutant had smaller visual-motor and optokinetic responses under light adaptation. Subsequent to dark adaptation, the two responses of mutant and wild-type fish were consistent, which suggested that rod cells were involved in the visual behavior response, even though they are immature in the early developmental stage (Venkatraman et al., 2020).

#### Variations of Visual Sensitivities

In different aquatic zones in nature, the spectral composition (color) and intensity (brightness) usually vary with depth due to light refraction, scattering, and absorption. In clean coastal waters, green light has a strong penetrating ability, while blue light can penetrate deeper into the water column. Dissolved organic matter and suspended particles in estuaries and freshwater areas cause light at shorter wavelengths to scatter and be absorbed, which also happens fast for yellow and red light, and is why coastal light appears typically greenish (Horodysky et al., 2013). Given differences in habitat and behaviors of aquatic organisms, visual sensitivity also varies among different types of animals.

Generally, nearshore benthic fishes are more sensitive to light intensity than pelagic fishes, but less sensitive than deep sea species. For instance, the benthic-dwelling flounder, *Paralichthys dentatus*, has greater luminous sensitivity than pelagic fish such as *Morone saxatilis*, *Pomatomus saltatrix*, and *Rachycentron canadum* (Horodysky et al., 2010; Horodysky et al., 2013). The spectral sensitivity and dynamic range of pelagic fishes are lower than those of nearshore fishes. Fish living in the same body of water at different depths may also have different spectral sensitivities, which are closely associated with the spectral composition of the marine environment. Several groups reported (Matsumoto et al., 2009; Horodysky et al., 2013; Mccomb et al., 2013) that the range of spectral sensitivities of *Chaetodipterus faber*, *Tautoga onitis*, and *Centropristis striata* living in temperate coral reefs was 400–600 nm. *Chaetodipterus faber*, which inhabits shallow water, tends to be more sensitive to the light (green) at longer wavelengths, whereas *T. onitis* and *C. striata*, which live in deep water, tend to be more sensitive to the light (blue) at shorter wavelengths.

Visual sensitivity is also associated with lifestyle. In the same habitat, species that are active at night generally have lower temporal resolutions, while species that are active during the day have higher temporal resolutions (Matsumoto et al., 2009; Mccomb et al., 2010). In low visibility environments, organisms may exhibit prolonged integration times of the retina, which means that temporal resolution is reduced to capture light to the greatest extent possible (Warrant, 1999; Kalinoski et al., 2014; Ryan et al., 2017). Matsumoto et al. (2009) used an exponential function to fit the frequencies of the flicker stimulations and ERG amplitude, treated the slope of the exponential function as the temporal resolution, and found that the dark-adapted temporal resolution of the tuna, *Thunnus orientalis*, was significantly lower than that of the mackerel, *Scomber japonicas*. The former tended to be more active during the day and was insensitive to light intensity, with the irradiance required to generate a half response of the maximum ERG amplitude being 1.38 quanta cm^–2^s^–1^. The luminous sensitivity of the perch, *Siniperca chuatsi*, which becomes accustomed to preying at night under light adaptation conditions, is much lower than that under dark adaptation; the irradiance at which the ERG signal appears under light adaptation is 1,000 times greater than that under dark adaptation (Liang et al., 1994).

Aquatic organisms also adapt to the environment by optimizing their visual system. Mccomb et al. (2010) found that three species of coastal sharks (*Sphyrna tiburo*, *Sphyrna lewini*, and *Carcharhinus acronotus*) had scotopic spectral sensitivity peaks that fit the spectral range of their environment during the high predation period at dusk. The temporal resolution of *T. onitis* and the diel cycle invariance of luminous and spectral sensitivities are consistent with this species’ dormant lifestyle at night (Horodysky et al., 2013). Lampreys are usually active at night, and their temporal resolution and contrast sensitivity correspond to their lifestyles and habitats. For example, the temporal resolutions of parasitic *Mordacia mordax* and *M. praecox* are lower than 50 Hz. Moreover, the temporal resolution of parasitic species at all temperatures and light intensities is higher than that of non-parasitic species, and their response to flickering square-wave white light stimuli at a frequency of 5 Hz decreases with decreasing contrast levels. As temperature increases, the contrast sensitivity of lampreys also increases, which suggests that ambient temperature restricts the visual function of poikilotherms (Warrington et al., 2017).

Most fish species exhibit a diel rhythm cycle with respect to their visual sensitivity (Bassi and Powers, 1987; Li and Dowling, 1998; Kunz, 2006). The b-wave and d-wave amplitudes of *D. rerio* juveniles (5 dpf) are normal during the day but substantially disappear at night. Even under total darkness, the amplitude is high during subjective days and low during subjective nights, which can be attributed to the fact that the activity of the photoreceptor outer segment decreases and synaptic ribbons in the cone pedicles disassemble (Emran et al., 2010). The ERG amplitude of the tarpon, *Megalops atlanticus*, also shows a diel rhythm cycle, with the highest visual sensitivity at midnight and the lowest at noon. Under continuous darkness, the visual sensitivity during subjective nights is significantly higher than during subjective days, which suggests that it may be directly regulated by an endogenous biological clock (Kopperud and Grace, 2017).

The visual sensitivity of fish also varies among different developmental stages. Visual sensitivity in the juvenile stage is lower than that in the adult stage. The peak wavelengths of dark-adapted spectral sensitivity in juvenile *T. orientalis* is 474–494 nm, and the luminous sensitivity tends to increase with growth stage (Matsumoto et al., 2011). Sexually mature three-spined sticklebacks (*Gasterosteus aculeatus*) show higher sensitivity to the red spectrum (Shao et al., 2014). The male cichlid, *Astatotilapia burtoni*, exhibits a change in body color during courtship. Compared with non-sexually mature females, the sexually mature female *A. burtoni* is more sensitive to wavelengths similar to those of the male’s body color during courtship. Subsequent to stimulative ovulation, females also have a higher visual sensitivity compared to pre-injection measurements (Butler et al., 2019).

#### Effects of Environmental Factors on Visual Sensitivity

The variation of environmental factors also impacts vision. Hypoxia reduces visual sensitivity. Stenslokken et al. (2008) reported that severe hypoxia irreversibly impaired the vision of the shark, *Hemiscyllium ocellatum*. Acute hypoxia affects the rods and cones of the goldfish, *Carassius auratus*, to varying extents; the effect of acute hypoxia on b-waves under light adaptation is faster than that on b-waves under dark adaptation, which suggests that the signaling pathway of cones tends to be more sensitive to hypoxia than the signaling pathway of rods (Wei et al., 1995). Temperature can also affect the sensitivity of the retina. For example, rapid cooling may damage the retina of the catshark, *Scyliorhinus canicula*, because the ERG waves do not appear after timely rewarming. The b-wave amplitudes of the carp, *Carassius gibelio*, increased significantly after rewarming, but it did not fully recover to the original value. However, the visual sensitivity of the eel, *Anguilla*, did not differ significantly as a function of temperature. Subsequent to rewarming, the b-wave amplitude exceeded the initial level (Gacic et al., 2015). The composition of feed also affects visual sensitivity. Compared with a group of bass (*Dicentrarchus labrax*) fed with 0% or 1.5% taurine, the peak of spectral sensitivity of *D. labrax* fed with 5% taurine gradually shifted to longer wavelengths. The light intensity required to reach 75% of the ERG amplitude peak differed significantly among fish fed with feed containing different amounts of taurine (Brill et al., 2019).

In aquaculture and fisheries, the effects of anomalous light on the visual sensitivities of aquatic organisms need to be considered. Strong light may affect the visual sensitivity of fish, and different light periods and wavelengths during the rearing process may also affect the fish growth. ERG can be used to assess the visual sensitivity trends of fish as they adapt to different background light conditions. Dixon et al. (2004) found that compared with natural light, continuous acclimation under blue, green, and orange light reduced the sensitivities of *D. rerio* juveniles (6–10 dpf) to ultraviolet stimuli. Continued dark rearing also reduced the visual sensitivity of *D. rerio* and affected early visual development (Saszik and Bilotta, 1999). However, the negative effects from rearing under dark conditions resumed after placing *D. rerio* under normal light (Saszik and Bilotta, 2001). The retina is also susceptible to light during development, but its structure and function show a certain plasticity. The peak of spectral sensitivity of *M. atlanticus* adapted to red light (at long wavelengths) for 2–4 months was significantly different from that of the fish adapted to blue light (at short wavelengths); subsequent to dark adaptation, the irradiance required for light stimuli to achieve a response was less than that of light adaptation (Schweikert and Grace, 2018). The frequency and intensity of light stimuli also affect fish behavior. After strobe light stimuli, the visual sensitivity of the carp, *Hypophthalmichthys molitrix*, and *H. nobilis* was reduced (Vetter et al., 2019). After exposure to strong light (approximately 2,000 μmol⋅m^–2^⋅s^–1^) for 15 min, the visual sensitivity of the halibut, *Hippoglossus stenolepis*, decreased (Brill et al., 2008), and the decrease may have been irreversible (Magel et al., 2017).

### Application of Electroretinographys in Studies of Crustacean Visual Function

#### Types of Photoreceptors

Photoreceptors are evolutionarily classified into ciliary and rhabdom types according to the structural characteristics of their membranes. There are various types of invertebrate photoreceptor systems; primitive types include the ocellus of protozoa, and more developed types include the camera eyes of cephalopods. Despite structural differences, their cells consist of three parts: a photosensory part, cell body, and axon (Huang, 1988). In crustaceans, photoreceptors are monocular and compound. Compound eyes are composed of the ommatidium, and its outside to inside structure consists of a refraction system, photosensitive system, and pigment cells. In the photosensitive system, the protruding microvilli of the retinula cells comprise the rhabdom. The structure of crustacean eyes is also associated with light intensity. Light and dark adaptation trigger changes in the ultrastructure of eyes, the most obvious being migration of pigments and changes in the position, size, and shape of the rhabdom. After light adaptation, pigment particles are distributed across the photoreceptor cells, the diameter of the rhabdome shrinks, and the microvilli arrangement is disordered; after dark adaptation, the diameter of the rhabdome enlarges, the microvilli arrangement is regular, and the pigment particles are only distributed at the distal and proximal ends of cells (Meyer-Rochow, 1999, 2001; Matsuda and Wilder, 2014).

#### Factors Impacting Visual Sensitivity

The visual sensitivity of crustaceans is closely associated with habitat. Analogous to the absorbance characteristics of rhodopsin, crustacean spectral sensitivity is modified by the attenuation of light by the dioptric apparatus and the length and absorption coefficient of the rhabdom (Johnson et al., 2002). Unlike direct measurement of the absorbance of the visual pigment, total response of the retinal photosensitive layer to light stimulation, as measured by ERG, considers the light filtering of other eye parts (Bryceson, 1986). Nearshore species are generally more sensitive to light at long wavelengths than oceanic species, and the peak of spectral sensitivity of shallow water species is different from that of deep sea species, which coincides with the spectral composition of the habitat. The shrimp species, *Neomysis integer, Praunus flexuosus*, and *P. inermis*, are offshore species, and their peak wavelengths of spectral sensitivity are 515, 530, and 535 nm, respectively; *Mysis mixta* and *M. relicta* sp. II are pelagic species, and their peak values are 510 and 520 nm, respectively (Lindstrom, 2000). Three shallow-water decapods (*Crangon allmani, Pandalus montagui*, and *Nephrops norvegicus*) are most sensitive to light in the range of 510 to 525 nm, whereas the peaks of spectral sensitivity of two deep sea decapods [*Paromola cuvieri* and *Chaceon* (*Geryon*) *affinis*] are below 500 nm (Johnson et al., 2002). The peak of spectral sensitivity of deep sea creatures is generally in the blue region of the visible spectrum. Frank et al. (2012) used ERG to identify the spectral sensitivity of eight benthic crustaceans and found that the spectral sensitivity peaks ranged from 470 to 497 nm. The squat lobsters, *Euumunida picta* and *Gastroptychus spinifer*, are also sensitive to light at ultraviolet wavelengths, which may be associated with deep sea bioluminescence.

Differences in spectral sensitivity are also closely associated with phylogeny. Under the same spectral distribution, Audzijonyte et al. (2005) found that the peak wavelength of spectral sensitivity of the lake species, *M. relicta* (564 nm), was higher than that of bay species, *Mysis salemaai* (545 nm). The spectral sensitivity of *M. relicta* collected from four geographically isolated areas also differed, and the difference was not related to the spectral distribution, but rather to the geographic location (saltwater/freshwater). Donner et al. (2016) examined the visual sensitivities of three species of mysids (*M. relicta*, *M. salemaai*, and *Mysis segerstralei*) and reported that the peak spectral sensitivity of lake populations was greater than that of brackish water species, and that this difference had minimal effects from the light environment. As the light intensity in a habitat becomes weaker with increasing depth, the luminous sensitivity of crustaceans increases and the temporal resolution tends to decrease, but there are some exceptions. Some species that are distributed in deeper zones have a much higher temporal resolution. For example, the CFFs of krill (*Nematobrachion flexipes* and *N. sexspinosus*) distributed at 400–600 m were 44 and 56 HZ, respectively, which may be due to the bioluminescence of most of the live prey (Frank, 1999, 2000; Johnson et al., 2000). The temporal resolution of crabs is generally lower than that of shrimp. Of the collected benthic crustaceans in their study, Frank et al. (2012) found that the CFFs of two shrimp, (*Heterocarpus ensifer* and *Eugonatonotus crassus*) were within the range of 16 to 24 Hz, whereas the CFFs of four crab species (*G. spinifer, E. picta, Munidopsis erinacea*, and *Bathynectes longipes*) were slightly lower (10–14 Hz), presumably due to the less mobile lifestyle of crabs.

Like most fish species, strong light stimuli reduces the electroretinogram saturated amplitude of crustaceans, which is associated with the intensity and duration of light stimuli. However, an animal’s visual sensitivity can recover to a certain extent. A moderate increase in adaptation to strong light over a long period of time will relieve the damage caused by strong light stimuli, and this process is associated with the rate at which light intensity increases (Viljanen et al., 2017). For two populations of *M. relicta*, the peak amplitude of ERG decreased after 2–3 weeks of strong light exposure, and the sensitivity to light intensity recovered at a slow rate under dark adaptation. Based on these results, the researchers suggested that recovery of retinal sensitivities of marine populations is limited by the regeneration rate of 11-*cis* retinal, whereas that of retinal sensitivity of lake populations is limited by the photoreceptor membrane turnover (Feldman et al., 2020).

#### Diel Cycle Changes in Visual Sensitivity

The visual sensitivity of crustaceans also follows a circadian rhythm. For example, Cai and Zheng (1990) found that the luminous sensitivity of the crab, *Orithyia sinica*, followed a circadian rhythm, whereas the spectral sensitivity curves and peak values during the day and at night generally remained the same. The luminous sensitivity of the crab, *Poriunus trituberculaius*, and the prawn, *Penaeus japonicas*, showed no change with circadian rhythm, and the spectral sensitivity curves and peak values during the day and at night were perfectly matched. These differing results may be associated with changes in light conditions in the animal habitat. In another study, ERG analyses were performed on the retina and laminar ganglion of the eyestalk of the crayfish, *Procambarus clarkia*. The luminous sensitivity of the two tissues during the day was lower than that at night, with a cycle approximately 22–23 h. When day was treated as a dark condition and night was treated as a light condition, the ERG waves at night remained higher than those during the day (Aréchiga and Rodríguez-Sosa, 1998). Pigment dispersion hormone (PDH), which functions as a non-photosynchronizer, can synchronize the circadian rhythm of *P. clarkia*. Studies showed that injection of PDH into an isolated eyestalk could advance or delay the circadian response of its visual photoreceptor depending on the time of treatment (Verde et al., 2007; Solis-Chagoyan et al., 2012a). Hormones can depolarize the potential of the photoreceptor membrane receptors. In a diel cycle, the light sensitivity varies from photoreceptor to photoreceptor. When the eyestalk isolated from *P. clarkia* was injected with PDH during the subjective day, ERG activation was slow, with a longer latency (the duration from light stimuli to the time when the ERG signal reached 10% of the peak amplitude) and half-time of activation (the duration from the time when the ERG signal reached 10% of the peak amplitude to the time when it reached 50% of the peak amplitude) when compared with the results for subjective night (Barriga-Montoya et al., 2010). Barriga-Montoya et al. (2017) reported that when subjected to light stimuli, the recovery of visual sensitivity was associated with a circadian rhythm, and the recovery of visual sensitivity with PDH depended on the duration of the circadian rhythm.

The amplitude at which photoreceptors show an electric response to light is also affected by melatonin, and the release of melatonin generally shows a circadian rhythm. At a low concentration of endogenous melatonin, the ERG amplitude increased when *P. clarkia* was injected with melatonin, and this induced effect was similar to the ERG response level observed at a high concentrations of endogenous melatonin (Solis-Chagoyan et al., 2012b). Melatonin synchronizes the cycle and phase of the circadian rhythm, thus the use of exogenous melatonin at different times may have specific effects on the rhythm phase. The phase advances and slows during a subjective day, but there is no phase change during a subjective night (Solis-Chagoyan et al., 2008).

### Application of Electroretinography in Studies of Mollusk Visual Function

#### Types of Photoreceptors

Most photosensitive organs of mollusks are less structurally organized, but cephalopods have highly developed camera-type eyes. With a simpler retina structure compared to vertebrates, the retina of cephalopods consists of a rhabdom layer, melanin layer, optic cell nuclear layer, and nerve fiber layer. There is only one type of photoreceptor cell (rod cells), and two visual pigments are present in the cephalopod retina: rhodopsin in the outer segment of the photoreceptor-type cell and retinal pigment in the myelin corpuscle membrane of the inner segment. Due to the lack of color-differentiating cone cells, cephalopods are generally considered to be color blind. Scallops also have complex photosensitive organs. In their typical mirror eye, the retina is divided into a distal and a proximal retina. The distal retina consists of ciliary photoreceptor cells, which are homologous to rods and vertebral cells of vertebrates; the proximal retina consists of microvilli photoreceptor cells, which are homologous to those of invertebrates (Speiser et al., 2011; Sun, 2015). The retina of gastropods contains some photoreceptor cells with microvilli and cilia. Electrophysiological measurements revealed that the spectral sensitivity of various gastropods only had a single peak, with a wavelength range of 475 to 496 nm (Gillary, 1974; Chernorizov et al., 1992, 1994; Zhukov et al., 2006; Shepeleva, 2013). Gao et al. (2016c) used histological methods to divide the eye tissue of the abalone, *Haliotis discus hannai* from outside to inside into a retinal pigment epithelial cell layer, outer nuclear layer, photoreceptor inner segment, inner nuclear layer, melanin particle deposition layer, and optical fiber layer.

#### Measurement of the Spectral Sensitivities of Mollusks

Hamasaki (1968a) anesthetized octopus (*Octopus vulgaris* and *O. briareus*) samples for ERG analysis and found a continuous negative wave on the recorded electroretinogram. The amplitude and light adaptation state were associated with light stimuli intensity; as the stimuli intensity decreased, the negative wave amplitude decreased and the latency of the response increased. Under dark adaptation or with adaptation to different spectral components, the peaks of spectral sensitivity were approximately 480 nm, which suggested that there was only one visual pigment in octopus (Hamasaki, 1968b). The peak of spectral sensitivity of the cuttlefish, *Sepiella maindroni de Rochebrune*, appeared at 490 nm when the ERG recording was performed with adaptation to background light at different intensities and wavelengths, but the peak did not shift and the curve shape stayed the same (Zheng and Cai, 1981). Yamamoto et al. (1985) classified the process of *Sepiella japonica* development from egg laying to hatching into 40 stages. ERG readings were first observed at Stage 34 when the photoreceptor cells began to differentiate into inner and outer segments. From Stage 35 to 36, the density and arrangement of microvilli tended to be more regular, leading to the formation of the rhabdome and an increase in the ERG amplitude; by Stage 39, the ERG amplitude reached the individual adult level. In another study, the ERG amplitude of *Sepia officinalis* decreased as the body size increased, and its sensitivity to blue light was 100 times greater than that of yellow light (Groeger et al., 2006).

The squid, *Euprymna scolopes*, has a bioluminescent organ containing symbiotic photobacteria (*Vibrio fischeri*), which has a photosensitive function. In an ERG test, this organ generated a positive amplitude signal under light stimuli, which was opposite the negative amplitude generated from eyes under light stimuli. However, this difference was not associated with the presence or absence of photobacteria. The ERG amplitude of photogenic tissue was only associated with the size of the bioluminescent organ; the larger the photogenic organ, the greater the response amplitude (Tong et al., 2009). Spectral responses of the scallop *Amusium japonicum* spanned 433–700 nm, with a peak of approximately 470–520 nm. These values indicate that this organism is well-adapted to the light environment of a shallow sea habitat. Its CFF was 1.3–1.5 Hz, indicating that only slow-moving objects could be detected (Kanmizutaru et al., 2005). The range of spectral sensitivities of the larvae of the oyster, *Magallana gigas*, is 500 to 650 nm, with a peak at 620 nm (Kim et al., 2021). As a typical diurnal nocturnal creature, the movement and feeding behaviors of abalone follow an obvious circadian rhythm (Gao et al., 2020). However, Gao et al. (2016d) found that abalone showed phototaxis toward darkness (red and orange light at long wavelengths) but hide from blue and green light at short wavelengths. Under blue and green light, the growth, survival, and feed conversion ratio of abalone were significantly lower than those under red and orange lights (Gao et al., 2016b). Under blue and green lights at short wavelengths, the larval metamorphosis and survival rate of *H. discus hannai* were significantly higher than those under red and orange lights (Gao et al., 2016a,^2017^). This finding suggests that in diverse stages of growth and development, abalone are sensitive to different spectral components to a varying extent, and therefore electrophysiological technology may provide the best way to assess the spectral sensitivity of abalone.

Certain gastropods are able to regenerate their eyes. For example, the ERG results of retina from a regenerated eye of the snail, *Achatina fulica*, with or without morphological abnormality, was similar to that of a normal eye. In different age groups, the ERG signals of regenerated eyes resembled, but were all slightly lower than, those of normal eyes. Under recurring stimuli, the amplitudes of the signals from regenerated eyes decreased, but the extent of the amplitude was greater than that of a normal eye. Additionally, during the period of recovery, the amplitude of the response decreased with age (Flores et al., 1983; Tartakovskaya et al., 2003).

### Application of Electroretinography in Studies of Visual Functions of Other Aquatic Animals

When spectral sensitivity is measured by flash-ERG, it usually takes a long time to record. During this period, the amplitude of ERG components may be subject to various parameters, such as changes in the eyes, the electrode position, or the metabolic state of the retina. Another alternative is to use flicker-ERG, in which the intensity of the monochromatic test light is adjusted to generate an ERG amplitude identical to the fixed reference light stimuli. This alternative has some strengths when used for spectral sensitivity measurements: any ERG change that occurs during the test may also be reflected in the response to the test and reference lights. In addition, it only requires one measurement for each specific test wavelength, thereby shortening the test duration (Neitz and Jacobs, 1984; Jacobs et al., 1996).

When the spectral sensitivity of sea turtles was measured by flicker-ERG, at least two visual pigments were found in adult *Dermochelys coriacea*, *Caretta*, and *Chelonia mydas*, which were sensitive to the longer wavelengths, with peaks at 580 nm. *Dermochelys coriacea* showed higher sensitivity in the short wavelength region, with a peak at 500 nm. In contrast, the ERG results of *C. mydas* and *C. caretta* had a secondary peak at approximately 520 nm. When *C. caretta* and *D. coriacea* hatchlings were assessed using flicker-ERG, both species had peak sensitivities between 520 and 540 nm and a secondary peak at UV wavelengths was detected, but the sensitivity of *D. coriacea* to light at wavelengths higher than 520 nm was significantly lower than that of *C. caretta* (Levenson et al., 2004; Crognale et al., 2008; Horch et al., 2008).

Color vision is based on the extent to which the visual system can respond to light at different wavelengths, and it is based on two or more photoreceptor types. Most marine mammals have no color vision. For example, the seal, *Phoca vitulina*, only has rods and only a few cones, so it likely is color blind. Compared to terrestrial mammals, marine mammals lack cones sensitive to blue light, which may have resulted from prolonged adaptation to the marine environment (Peichl and Moutairou, 1998; Griebel and Peichl, 2003). This also coincides with the electrophysiological results for *P. vitulina*. The flicker-ERG test results revealed whether it was adapted to different spectral conditions, with the ERG amplitude having only one peak at 510 nm (Crognale et al., 1998). Levenson et al. (2006) used flicker-ERG at different frequencies and found that its peak of spectral sensitivity was at 502.5 nm, where rod cells play a dominant role. However, early behavioral studies suggested that the sea lion, *Zalophus californianus*, could identify color, which was supported by comparison of cone signals and rod signals (Griebel and Schmid, 1992; Griebel and Peichl, 2003). However, Scholtyssek et al. (2015) conducted follow-up behavioral studies and maintained that previous experiments may have neglected the effects of brightness and contrast, so *Z. californianus* may identify color based on brightness. Oppermann et al. (2016) conducted behavioral studies to show that *Z. californianus* could identify different colors, and further inferred that the low light intensity used by Scholtyssek et al. (2015) (in which cones cannot function) may explain their conclusion that *Z. californianus* is color blind.

Electroretinography has also been used to detect visual sensitivity of jellyfish. The form of the crystallin lens eyes of *Tripedalia cystophora* resembles that of vertebrates and cephalopods. Crystallin lens eyes include lower and upper lens eyes, and both have similar spectral sensitivities, with the peak corresponding to blue and green light at a wavelength of 500 nm (Coates et al., 2006). The visual system of the horseshoe crab, *Limulus Polyphemus*, exhibits a circadian rhythm. Under the natural light cycle, the ERG results showed that the visual sensitivity rhythm of *L. polyphemus* coincided with the circadian cycle. Even when *L. polyphemus* was fed in darkness for 1 year, its visual sensitivity followed the circadian rhythm. The circadian rhythm in subjective days was higher than those in subjective nights, and the rhythmic phase of the electroretinogram revealed an advance or delay of phases depending on exposure to short durations of different lighting conditions (Barlow, 1983; Watson et al., 2008). Information about components of the visual system of an increasing number of aquatic organisms is becoming available, and ERG can be used to further identify retinal functions, study how aquatic organisms adapt to different habits, and determine how to protect endangered species.

## Prospects

This review focused on the use of ERG in studies of visual sensitivities of aquatic organisms. ERG can accurately reflect the response of the retina to light, and data acquisition is quick and precise. The variations of ERG waveforms caused by different light stimuli are extremely useful for observing the visual function characteristics of aquatic organisms, including spectral sensitivity, luminous sensitivity, and temporal resolution. Considering the enormous differences in body size, eye size, and form of aquatic organisms, the first step when studying a species is to reasonably reconstruct the characteristics of the eyes. For example, electrodes that can tightly fit the eyes and record data with a high signal-to-noise ratio should be used. Second, information about the general composition of visual organs and the retinal structures of aquatic organisms is incomplete, so there is an urgent need to describe the basic parameters, such as visual formation stages, retinal structure, and cell type and function, using anatomy, histology, and scanning or transmission electron microscopy. These parameters are crucial elements of ERG analysis. Third, the parameters of ERG light stimuli should be selected so that they can be adjusted according to the species being studied. The International Society for Clinical Visual Electrophysiology has published a set of standards for clinical ERG, but they are derived from human clinical research. Limited data are available for aquatic animals. To effectively assess the function of cone or rod cells, the durations of light and dark adaptations must be considered, the duration of flicker stimuli should be shorter than the integration time of photoreceptors, and the interval of stimuli must not affect the current state of retinal light adaptation. Fourth, ERG records the general response of retinal cells to light, but it is still difficult to associate the changes of a given ERG pattern with the specific changes of retinal light responses. Each waveform of the electroretinogram reflects the activity of different retinal cells, and wave components formed may overlap to some extent, thereby undermining the process of analyzing the functions of individual cells based on their unique wave components. To investigate the functions of specific retinal cells, efforts should be made to block the expression of certain neuronal functions using drugs, or to use models with loss of functions or mutations to isolate the corresponding wave components. We also need to develop a technique to accurately analyze the ERG waveform and associate the derived parameters with the activity of retinal cells in order to better describe the function of the retina. Finally, based on the retinal structure identified in aquatic organisms, the visual acuity of species dwelling in the same habitat could be inferred from the determination of ERG curves, and from comparisons and analyses of the characteristics of ERG curves of sibling species. This will, in turn, provide immediate guidance for the optimization of light conditions in aquaculture practice and the selection of lamps in the marine fishing industry.

## Author Contributions

XG and CK conceptualized the study. SL, MZ, and ML conducted research and collected the data. YL, WY, and XL provided the materials and the device. SL and XG wrote the manuscript. CK had primary responsibility for the final content. All authors read and approved the final manuscript.

## Conflict of Interest

The authors declare that the research was conducted in the absence of any commercial or financial relationships that could be construed as a potential conflict of interest.

## Publisher’s Note

All claims expressed in this article are solely those of the authors and do not necessarily represent those of their affiliated organizations, or those of the publisher, the editors and the reviewers. Any product that may be evaluated in this article, or claim that may be made by its manufacturer, is not guaranteed or endorsed by the publisher.
